# The Role of circRNAs in the Pathological Mechanisms of Alzheimer's Disease: Potential Biomarkers for Diagnosis

**DOI:** 10.2174/011570159X337659241014140824

**Published:** 2024-10-24

**Authors:** Zulalai Abuduwaili, Yingao Fan, Wenyuan Tao, Yanting Chen, Yun Xu, Xiaolei Zhu

**Affiliations:** 1 Department of Neurology, Nanjing Drum Tower Hospital, Affiliated Hospital of Medical School, Nanjing University, Nanjing 210008, China;; 2 State Key Laboratory of Pharmaceutical Biotechnology and Institute of Translational Medicine for Brain Critical Diseases, Nanjing University, Nanjing, China;; 3 Jiangsu Key Laboratory for Molecular Medicine, Medical School of Nanjing University, Nanjing, Jiangsu, China;; 4 Jiangsu Province Stroke Center for Diagnosis and Therapy, Nanjing, Jiangsu, China;; 5 Nanjing Neuropsychiatry Clinic Medical Center, Nanjing, Jiangsu, China

**Keywords:** Alzheimer's disease, circular RNAs, pathological mechanisms, biomarkers, therapeutic targets, dementia

## Abstract

Alzheimer's disease (AD) is the most common neurodegenerative disease leading to dementia in the elderly, and the mechanisms of AD have not been fully defined. Circular RNAs (circRNAs), covalently closed RNAs produced by reverse splicing, have critical effects in the pathogenesis of AD. CircRNAs participate in production and clearance of Aβ and tau, regulate neuroinflammation, synaptic plasticity and the process of apoptosis and autophagy, indicating that circRNAs may be alternative biomarkers and therapeutic targets. Our review summarizes the functions of circRNAs in the progression and development of AD, which provide insights into the prospect of circRNAs in the diagnosis and treatment of AD.

## 1 INTRODUCTION

As a progressive and irreversible neurodegenerative disease, Alzheimer's disease (AD) causes most dementia and results in memory loss and cognitive impairment, affecting millions of people in the whole world. In the United States, 6.7 million people over 65 years old suffer from AD, and the number will double by 2060 unless there is a medical breakthrough in AD. AD has undoubtedly brought a huge economic burden and pressure on individuals and the whole society [1].

Early-onset AD (EOAD) and late-onset AD (LOAD) are the two main forms of AD. EOAD generally occurs before the age of 65, accounting for 1% to 5% of all AD [2]. The genetic mutations of EOAD mainly include genes of amyloid precursor protein (APP), presenilin 1 (PSEN1) and presenilin 2 (PSEN2) [3], leading to protein proteolysis of APP to produce a mixture of Aβ peptides and aggravate amyloid plaques [4]. ApoE ε4 remains the major genetic risk factor in LOAD, and the ɛ4 allele can raise the disease risk and lower the age of onset [5]. In addition, multiple genes have been identified in LOAD, including CD33, the ATP-Binding Cassette Subfamily A Member 7 gene (ABCA7), triggering receptor expressed on myeloid cells 2 (TREM2) and bridging integrator 1 (B1N1), which are involved in oxidative homeostasis, metabolism of protein and cholesterol as well as synapse function [6].

**Fig. (1):**
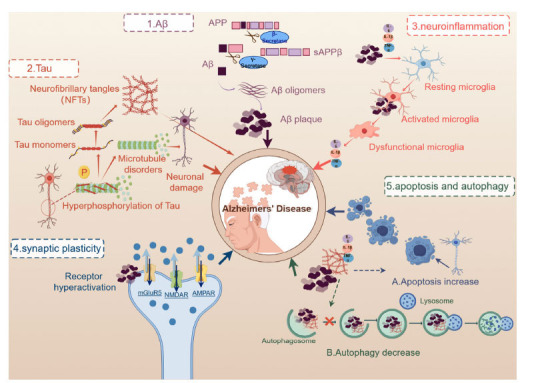
The key pathological features of AD. **1**. The process of generating Aβ: β-secretase first cleaves APP into sAPPβ and β-C-terminal, and then γ-secretase cleaves it to produce Aβ peptide fragments. Aβ aggregates into oligomers, which then progressively form amyloid plaques. **2**. The process of Tau: tau proteins are highly phosphorylated and detach from microtubules, leading to microtubules disorganization and damage to neurons. Phosphorylated tau proteins accumulate and form neurogenic fiber tangles (NFTs), which have toxic effects and lead to AD. **3**. Neuroinflammatory processes: microglias activated by Aβ and inflammatory factors (IL-6, IL-1β, TNF-α) can ingest and degrade Aβ, and chronically activated microglial cells become dysfunctional and lose their role in phagocytosis of Aβ, as well as releasing large amounts of inflammatory factors. **4**. Disruptions in synaptic plasticity: Aβ over-activates mGluR5, AMPA and NMDA receptors, leading to an imbalance between excitatory and inhibitory neuronal functions, resulting in synaptic damage. **5.A**. Excessive increase in neuronal apoptosis: Aβ and tau deposition, and inflammation can trigger the apoptotic pathway; **5.B**. Decreased autophagy in the brain: autophagy is defective in AD, which reduces Aβ and Tau clearance.

The pathogenesis of AD has not been fully defined. The accumulation of beta-amyloid (Aβ) is the core of pathological processes, known as the “amyloid cascade hypothesis” [7]. Overproduction or decreased clearance of soluble Aβ will induce its self-assembly into oligomers, which eventually form insoluble amyloid plaques [8]. In addition, Aβ induces hyperphosphorylation of tau protein, neurofibrillary tangles (NFTs), neuroinflammation and oxidative stress. However, emerging evidence suggests that these pathological features may occur independently of the initial Aβ trigger [9]. Eventually, Aβ deposition, Tau tangles, synaptic dysfunctions, abnormal neuronal apoptosis, autophagy and neuroinflammation will be fundamental to the preclinical and clinical phases of AD [10] (Fig. **1**).

**Fig. (2):**
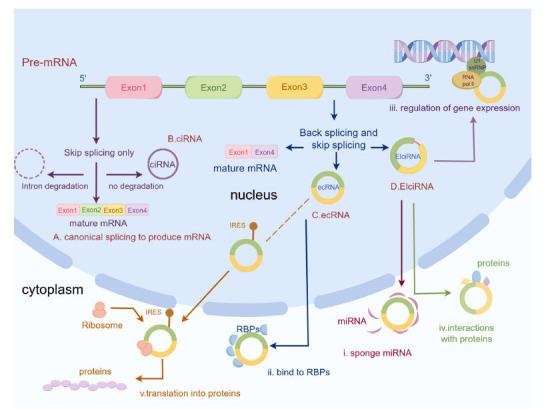
Splicing models and functions of circRNAs. Splicing models of circRNAs: (**A**) Classical pathway: pre-mRNA splicing forms mature linear mRNA, exons link and introns dissolve. (**B**) Introns are not solubilized but reverse spliced to form intron-only circRNAs (ciRNAs). (**C**) The pre-mRNA is reverse spliced to form circRNA: the 5' splice site downstream of the intron is joined to the upstream 3' splice site to form a circular RNA. Intron-free circRNAs(ecRNA) can be formed and the exons that are sheared off can form new mRNAs. (**D**) CircRNAs with both exons and introns (ElciRNA) can be formed when introns are retained. However, the functional and mechanistic differences between these circRNAs are not fully understood. Functions of circRNAs: (**i**) CircRNAs sponge miRNA and affect the expression of miRNAs. (**ii**) CircRNAs bind to RBPs and isolate them from their targets or modulate their expression. (**iii**) CircRNAs can regulate the expression of genes at the level of transcription by interacting with U1 snRNP and RNA polymerase II complex. (**iv**) CircRNAs can act as scaffolds, transporters or protein baits, but also can modulate protein activities by combining with them. (**v**) CircRNAs can be translated into proteins.

Circular RNAs (circRNAs) are one of the recently identified categories of non-coding RNAs (ncRNAs) which is produced by reverse splicing and the 3'-end of the transcript is spliced covalently with the 5'-end, creating a continual loop with covalently closed ends [11]. CircRNA molecules lack 3′ and 5′ termini, which makes them resistant to exonuclease degradation, and the mean half-life of circRNA is assumed to be at least 4-5 folds that of mRNAs [12]. Because of the covalently closed structures, they remain evolutionarily conservative with tissue-specific expression and can be regulated independently of their linear counterparts [13]. Various splicing models have been found [14], and the functions are summarised (Fig. **2**):

1. MiRNA sponging: Many circRNAs have miRNA recognition elements (MREs), then they can act as effective “sponges” *via* interactions with miRNA-Ago2 complexes and affect the expression of miRNAs’ targets [15].
2. Bind to RNA-binding proteins: CircRNAs can bind to RNA-binding proteins (RBPs), except for miRNAs, and isolate them from their targets or modulate their activity and ^persistence^, while RBPs can also regulate circRNAs’ expression [16].
3. Direct regulation of gene expression: CircRNAs have been found in the nucleus and they can regulate the expression of genes at the level of transcription by interacting with U1 small nuclear ribonucleoprotein (U1 snRNP), which can enhance RNA polymerase II complex function, or recruiting methylcytosine dioxygenase TET1 to promoter regions [17].
4. Interactions with proteins: CircRNAs can not only act as scaffolds, transporters or protein baits, but also modulate protein activities by combining with them [18].
5. Translation into proteins: Although circRNAs are generally considered as “noncoding” molecules, mass spectroscopy (MS) has uncovered plenty of translatable circRNAs [19]. Cytoplasmically localized circRNAs that have internal ribosome entry site (IRES) elements and AUG sites can be translated by CAP-independent mechanisms and N6-methyladenosine (m6A)-modifications may have a regulatory role in circRNAs translational activity [20].

Accumulating evidence suggests that compared to other tissues, circRNAs are abundantly expressed in the brain [21]. Besides, most circRNAs are enriched in synapses, including presynaptic activity regions, presynaptic membrane and postsynaptic density. Since synapse dysfunctions have a vital role in cognitive impairment, it is hypothesized that circRNAs might be involved in neuronal activity and modulate cognitive functions [11], participating in cognitive-related diseases, such as AD.

## 2 CircRNA IN AD PATHOGENESIS

### 2.1 CircRNA and Aβ

The amyloidogenic pathway of APP leads to the production of neurotoxic Aβ. Typically, there is a balance between the degradation and production of Aβ, but during the pathology of AD, familial AD associated mutations lead to overproduction of Aβ, and the clearance of Aβ is impaired due to the dysfunction of glia cells. Multiple circRNAs participate in the Aβ pathology.

#### 2.1.1 CircHDAC9 and Aβ

MiR-138 is abundant in brains and elevated in the cerebrospinal fluid (CSF) of AD patients, acting as a negative regulator of dendritic spine morphogenesis [22]. In APP/PS1 mice, miR-138 is found to be age-dependently increased and promote the Aβ production *via* inhibiting the expression of a major α-secretase, a disintegrin and metalloproteinase domain-containing protein 10 (ADAM10) [23]. In addition, miR-138 has a highly conserved binding site on the 3′-UTR of Sirtuin 1 (Sirt 1) mRNA, and reduces the expression of Sirt 1, which directly activates ADAM10 transcription. CircRNA HDAC9 (circHDAC9) is found to have a binding site for miR-138 and serve as a miR-138 sponge. CircHDAC9 reverses the Sirt 1 inhibition and Aβ overproduction induced by miR-138 *in vitro*. Furthermore, in mild cognitive impairment (MCI) and AD patients, circHDAC9 is reduced in the serum, which probably increases the level of miR-138 and thus causes the aggravation of Aβ accumulation.

This research provides us with circHDAC9/miR-138/Sirt1 signal pathway for better understanding Aβ pathology in AD, indicating that it can be used as a therapeutic target in AD.

#### 2.1.2 Circ-0007556 and Aβ

Recent reports have shown that circRNA open reading frames (ORFs) can serve as templates for the biosynthesis of proteins. The circ-0007556 (circAβ-a) sequence contains the Aβ-coding region of the APP gene and can be translated into Aβ associated protein (Aβ175) in human embryonic kidney 293 (HEK293) cells and human brain samples. Aβ175 includes cleavage sites for β and γ secretases, implying that the Aβ peptide can be generated from Aβ175 and providing an alternative way of Aβ production *in vivo* [24]. However, circ-0007556 is extensively expressed among 33 circRNAs derived from the APP gene and is reduced in the entorhinal cortex of AD patients, with a negative correlation with Aβ depositions [25]. Given that at least 17 miRNAs are identified to target circ-0007556 [26], we speculate that the complex modulation system might contribute to the inconsistent results. What’s more, the circ-0007556 participation in pathological changes of AD probably depends on its translational mobilization but not on variation in the expression level.

All human individuals generate circ-0007556, which suggests that it may contribute to the pathogenesis of sporadic AD. Thus, the mechanisms of circ-0007556 in AD need to be confirmed by further research.

#### 2.1.3 CircCwc27 and Aβ

CircCwc27 is found to be abundant in neurons compared to other tissues and is overexpressed in the brains of APP/PS1 mice as well as in the temporal lobe cortex and plasma of AD patients.

In the context of AD pathology, a positive correlation is observed between the expression of circCwc27 and the level of Aβ40 or Aβ42. Knockdown of circCwc27 can reduce soluble and insoluble Aβ40 and Aβ42, and markedly decrease the areas of co-localization of dystrophic neurites with Aβ. What’s more, circCwc27 knockdown is found to prevent a reduction of synapse-associated proteins and significantly decrease pro-inflammatory markers. These results suggest that circCwc27 is involved in neurodegenerative pathologic processes and cognitive function in AD.

RNA pulldown assay and mass spectrometry (MS) analysis show that circCwc27 functions *via* binding to RNA-binding proteins including Pur-α in the brains. Pur-α is highly expressed in neurons and directly binds to and alters the activity of promoters of genes that are dysregulated in AD, including APP, membrane metalloendopeptidase (Mme, a key driver of Aβ degradation), Ntrk1, Ppp1r1b, Drd1 and Lhx8 [27]. Since circCwc27 is predominantly present in the cytoplasm, binding of circCwc27 and Pur-α affects the nuclear translocation of Pur-α and decreases the recruitment of Pur-α to these prompters, which results in an increase of APP and a decrease of Mme.

This article demonstrates that circCwc27 can interact with RBP Pur-α to regulate AD-related genes, revealing an unexplored role of circRNA in AD and circCwc27 knockdown can be a treatment strategy.

#### 2.1.4 Circ-0004381 and Aβ

Circ-0004381 is reported to promote neuronal damage in Parkinson's disease and AD. Circ-0004381 is increased after Aβ treatment in hippocampal neurons, and down-regulation of circ-0004381 attenuates hippocampal neuron apoptosis, mitochondrial dysfunction and oxidative stress.

Additionally, the bioinformatics database reveals that miR-647 has PSEN1 and circ-0004381 binding sites. PSEN1, a catalysis subunit of the γ-secretase complex, is increased in AD mice and promotes the deposition of amyloid plaques. Knockdown of circ-0004381 increases the expression of miR-647 but suppresses the level of PSEN1, while inhibition of miR-647 can reverse the reduction of PSEN1 caused by circ-0004381 knockdown. These data indicate that circ-0004381 can sponge miR-647 to regulate PSEN1 level [28]. In addition, circ-0004381 inhibition reduces the production of inflammatory factors such as IL-1β, IL-6, and TNF-α, resulting in a decrease in the M1-type (pro-inflammatory phenotype) and an increase in the M2-type (anti-inflammatory phenotype) of microglia, which suggests that circ-0004381 may modulate Aβ processing by microglia conversion.

#### 2.1.5 CircPSEN1 and Aβ

Autosomal dominant AD (ADAD) occurs before 65 years of age and there is autosomal dominant inheritance in families of three or more generations. CircPSEN1 is produced by PSEN1 mutations, and circPSEN1 gene counts are dramatically increased in individuals with ADAD, while no significant differences in linear PSEN1 gene expression are observed.

The circPSEN1 species include circ-0008521, circ-0003848, and circ-0002564, and the biological functions of these circPSEN1 species have not been characterized. Twenty-six miRNAs are identified to target these three species of circPSEN1 [29], which contribute to the functions of circPSEN1 in AD. For example, miR-144-3p can target APP and inhibit its expression, and miR-433 can target janus kinase2 (JAK2) to moderate its inhibition of Aβ-induced neuronal viability.

In conclusion, circPSEN1 is an essential modulator of the Aβ pathway, particularly in AD, and the underlying processes must be investigated. Furthermore, circPSEN1 counts could be used as indicators to differentiate AD from normal people and AD that occurs sometimes.

### 2.2 CircRNA and Tau

Excessive or aberrant phosphorylated tau will lose the biological activity that promotes microtubule assembly, leading to disordered microtubule depolymerization and axonal transport, which causes neuronal degeneration and cell apoptosis in AD [30]. Hyperphosphorylated tau eventually forms neurofibrillary tangles (NFT), which is one of the main pathologies of AD. AD has been divided into six stages based on the degree of NFT involvement [31], and it is included in the diagnostic criteria for AD neuropathology [32]. Recently, it has been identified that circRNAs are essential in tau protein pathology.

#### 2.2.1 Tau circRNA and TAU

Under pathological circumstances, MAPT can misfold to NFT and paired helical filaments (PHF), an insoluble intracellular protein polymer.

MAPT pre-mRNA is found to be able to form circRNAs that can be translated. By reverse splicing exon 12 to either exon 7 (12→7) or 10 (12→10), MAPT generates two circRNAs (tau circRNA): 12→7circRNA and 12→10 circRNA. The 12→7circRNA has an in-frame start codon, and the 12→10 circRNA does not, while both of them lack the stop codons. Thus, these circRNAs can be translated for several times by the ribosome, and RNA editing of these tau circRNAs promotes their translation and tau tangle formation [33].

The majority of circRNAs rely on pre-mRNA structures imposed by specific intronic Alu elements, which are widely modified by the adenosine deaminase acting on RNA (ADAR) enzymes [34]. ADAR enzymes change AUA to AUI as start codons of 12→10 circRNA for translation, and inflammatory signalling will promote the activation of ADAR enzymes [35]. Furthermore, 12→10 circRNA is ten times more frequent in human brains than 12→7 circRNA. As a result, when exposed to these tau circRNAs, there will be a large increase in the creation of tau tangle and NFT, which is explained by the ADAR enzyme activity and 12→10 circRNA translation.

This study on tau circRNA provides a novel mechanism of tau deposition, and modification of ADAR enzyme activity or removal of these tau circRNA may be an alternative treatment for AD and tau-associated disease.

#### 2.2.2 Circ-002048 and Tau

By analysing blood samples collected from the GEO database, circ-002048 is found to regulate has-miR-422a, has-miR-4784, and the-miR-3944-3p, and downregulation of circ-002048 results in increased expression of these miRNAs.

Interestingly, the common target gene of these three miRNAs is adaptor-related protein complex 2, mu 1 subunit (AP2M1), and they may limit the expression of AP2M1 [36]. AP2M1 is engaged in Clathrin protein-dependent endocytosis (includes autophagy), and is negatively associated with NFT. The regulating of the circ-002048-miRNA-mRNA network may inhibit AP2M1 expression and elevate the level of proinflammatory cytokines that contribute to tau hyperphosphorylation.

Moreover, Glycogen synthase kinase-3β (GSK3-β) is hyperactive in the AD brain, and hyperactive GSK3-β promotes phosphorylation of tau protein amino acid residues thereby facilitating toxic tau formation. The GO analysis reveals a strong connection between AP2M1 and GSK3-β as well as MAPT, suggesting that AP2M1 is involved in the process of tau formation.

Therefore, circ-002048 can promote AP2M1 through sponging corresponding miRNAs and alleviate tau pathology, which may be related to GSK3-β.

#### 2.2.3 CircPCCA and Tau

The level of circ-PCCA is reduced in the CSF of AD individuals, and it inhibits miR-138 expression. MiR-138-5p is elevated in the brains of AD individuals and AD cell models. In HEK293/tau cells, miR-138 overexpression activates GSK-3β and promotes phosphorylation of tau. In addition, miR-138 binds to 3′-UTR of retinoic acid receptor alpha (RARA) mRNA to reduce RARA expression, which notably inhibits miR-138-induced GSK-3β activity and decreases tau phosphorylation [37].

Furthermore, in AD patients, increased expression of circ-PCCA is associated with a reduced severity of the disease and may serve as a stand-alone indicator of a lower risk of AD [38].

#### 2.2.4 CircAPP, circPSEN1, circMAPK9 and Tau

In samples collected at the Boston University Alzheimer's Disease Research Center (BU-ADRC), the expressions of 45 circRNAs are associated with clinical dementia score (CDR) and AD neuropathology, including Braak stage and CERAD neuritic plaque score. Among these circRNAs, circAPP, circPSEN1 and circMAPK9 are noteworthy.

CircAPP, circPSEN1 and circMAPK9 are regulated independently of their relevant linear transcripts. Oligomeric tau (oTau) induces the reduction of circMAPK9, circAPP and circPSEN1 rather than their linear mRNAs, which indicates that oTau can specifically regulate their expression [39]. It is noteworthy that circPSEN1 is elevated in AD disease, which may be related to the early or late stage of the disease. In the pathophysiology of AD, some circRNAs may have more sensitive responses compared to their linear mRNAs, contributing to early diagnosis of pathological changes.

Moreover, circAPP inhibition increases the level of miR-15-5p, which inhibits IGF1 expression in neurons to affect tau phosphorylation [40]. The increase in circPSEN1 may decrease the availability of miR-13, which attenuates tau hyperphosphorylation by stimulating USP30 down-regulation [41].

CircRNA’s function and expression are independent of their linear correlates and show higher sensitivity, perhaps circRNA alterations are more representative of the underlying essence of the disease.

#### 2.2.5 CircMAN2A1 and Tau

The expressions of 276 circRNAs are associated with the Braak NFT stage in postmortem samples of the internal olfactory and temporal cortex during AD progression. What’s more, during AD progression, the circRNA adenosine to inosine (A>I) RNA editing is increased approximately threefold, but the linear mRNAs remain unchanged.

Previous studies have shown that RNA editing strongly promotes circMAPT translation to produce tau proteins, indicating that circRNA can work as a template for protein production [33]. CircMAN2A1 is associated with AD progression and its expression has been shown to grow with increasing Braak stage, along with upregulation of their A > I RNA editing. Subsequent transfection experiments with ADAR1-2 showed that circMAN2A1 can be translated after RNA editing and produce new proteins. However, the protein function and potential catalytic features still await to be determined.

This result indicates that circRNA translation may be elevated in the later stages of AD and is positively correlated with the severity of tau aggregation. This point provides a new understanding of circRNAs translation, and the mechanism of facilitating translation deserves to be further explored.

### 2.3 CircRNA and Neuroinflammation

Neuroinflammatory response is another critical feature in the development of AD. It is generally considered that in the early stage of AD, microglia can ingest and degrade Aβ, while chronically activated microglia lose their ability in phagocytosis of Aβ, and release large amounts of inflammatory factors that exacerbate neuroinflammation in AD. In addition, neuroinflammation promotes NFT aggregation and Aβ production and aggravates cognitive dysfunction in AD patients [42].

#### 2.3.1 Circ-0005835 and Neuroinflammation

Upregulation of circ-0005835 level has been found in the serum of AD individuals and Aβ-treated cells. Knockdown of circ-0005835 reduces Aβ-induced neuroinflammatory cytokines release, such as IL-6, IL-1β and TNF-α in BV2 cells, and facilitates the proliferation and differentiation of neural stem cells (NSC) to neurons.

Moreover, miR-576-3p has complementary binding sites with circ-0005835. MiR-576-3p expression is downregulated in the serum of AD individuals and Aβ-treated cells, and the knockdown of circ-0005835 enhances the miR-576-3p expression [43]. Circ-0005835 knockdown can decrease inflammation and increase neuronal viability in AD, while miR-576-3p inhibition compromised these effects, which suggests that circ-0005835 promotes AD development by sponging miR-576-3p.

However, the downstream of miR-576-3p still needs further validation.

#### 2.3.2 Circ-AXL and Neuroinflammation

Circ-AXL is upregulated in the cellular AD model, increasing apoptosis and inflammatory factors, and circ-AXL inhibition shows neuroprotective effects, while inhibition of miR-328 compromises these effects. Furthermore, miR-328 is identified as a target of circ-AXL in the cellular AD model, and circ-AXL can suppress miR-328 expression [44]. Notably, miR-328 can directly bind to BACE1, which exacerbates neuronal damage and inflammation of AD, and inhibits its expression, contributing to neuroprotective effects.

Furthermore, it has been observed that circAXL increases inflammation in SK-N-SH cells and modifies cAMP activity. It could be a result of circAXL's ability to target miR-1306-5p, which in turn influences the expression of phosphodiesterase 4A (PDE4A), a crucial regulator of cAMP degradation rates [45]. The circAXL/miR-1306-5p/PDE4A pathway also provides a deeper understanding of AD mechanisms.

#### 2.3.3 Circ-HUWE1 and Neuroinflammation

Circ-HUWE1 is significantly increased, while miR-433 is downregulated in the serum of AD individuals and SK-N-SH cells treated by Aβ_1-40._ MiR-433 has an excellent diagnostic value for AD because it shows a positive correlation with Minimum Mental State Examination (MMSE) scores in AD patients [46].

Interestingly, circ-HUWE1 binds to miR-433-3p and reduces its expression. Downregulation of circ-HUWE 1 rescues the apoptosis, inflammatory response and decreased cell viability through deregulating the suppression of miR-433-3p.

Fibroblast growth factor 7 (FGF7) facilitates the progression of inflammation and contributes to the progression of AD. And it is a target of miR-433-3p, which can inhibit its expression. What’s more, overexpression of circ-HUWE1 inhibits the activity of the Wnt/β-catenin signalling pathway. Thus, circ-HUWE1 can modulate the expression of FGF7 by targeting miR-433-3p to promote inflammatory progression and inhibit the activity of the Wnt/β-catenin signalling pathway.

#### 2.3.4 Circ-0049472 and Neuroinflammation

Circ-0049472 is upregulated in the CSF and serum of AD individuals and Aβ-induced cells. Silencing of circ-0049472 partially reverses Aβ-induced elevation of inflammatory cytokines (TNF-α, IL-6 and IL-1β). In addition, miR-107 is a target of circ-0049472 and participates in the development of AD and regulates Aβ-induced neuronal damage [47]. MiR-107 is predicted to target Kinesin family member 1B (KIF1B) and attenuates Aβ-induced neurotoxicity by downregulation of KIF1B [48]. KIF1B is increased in the CSF and serum of patients with AD, and it can cause a decrease in mitochondrial activity, leading to neuronal dysfunction [49].

Therefore, circ-0049472 upregulation enhances KIF1B expression by sequestering miR-107, thereby inhibiting cell viability and facilitating apoptosis, inflammation and oxidative stress.

#### 2.3.5 CircLPAR1 and Neuroinflammation

CircLPAR1 expression is elevated, while the level of growth differentiation factor 15 (GDF-15) is decreased in the brains of APP/PS1 mice and Aβ-treated SH-SY5Y cells. GDF-15 is generated by inflammatory stress and has a critical role in regulating neuroinflammation. CircLPAR1 knockdown or GDF-15 overexpression can protect cells from neuroinflammation, oxidative stress and neuron apoptosis caused by Aβ.

Upstream frameshift 1 (UPF1) has binding sites for both GDF-15 and circLPAR1, and GDF-15 mRNA and protein are reduced after circLPAR1 overexpression, which is reversed by si-UPF1 transfection. Thus, circLPAR1 negatively regulates the GDF-15 expression through interaction with UPF1.

Moreover, GDF-15 upregulation attenuates neuronal impairment by enhancing the expression of sirtuin 1 (SIRT1) to promote activation of the E2-related factor (Nrf-2)/heme oxygenase-1 (HO-1) pathway. In summary, downregulation of circLPAR1 has been found to attenuate pathological features of AD and cognitive dysfunction *in vivo via* GDF-15/SIRT1/Nrf-2/HO-1 pathway [50].

### 2.4 CircRNA and Synaptic Plasticity

Synaptic plasticity is a kind of activity-dependent change in neuronal connectivity strength and is regarded as a vital foundation for learning and memory formation [51]. Synaptic dysfunction occurs before the Aβ deposition and NFT and is closely associated with memory decline in AD. Aβ has been shown to over-activate mGluR5, AMPA, and NMDA receptors, leading to an imbalance between excitatory and inhibitory neuronal functions. In addition, it induces the inhibition of LTP and enhancement of LTD, which results in the disruption of synaptic plasticity [52]. Synaptic plasticity enhancement has been shown to ameliorate cognitive decline in neurodegenerative disorders.

#### 2.4.1 CircHOMER1 and Synaptic Plasticity

The HOMER1 gene participates in synaptic plasticity and learning and memory functions. Circ-0006916 is originally characterized as a circRNA derived from the synaptic HOMER1 gene and is consistently reduced in the brains of AD individuals. Recently, circ-0006916 has been found to decrease in the anterior prefrontal cortex, inferior frontal gyrus and parahippocampal gyrus [53]. In addition, the expression of circ-0073127, another circHOMER1, is decreased in the entorhinal cortex of female AD patients. Interestingly, the circHOMER1 changes are mainly found in the female group. Overexpression of circ-0006916 ameliorates Aβ_42_-induced neuronal damage by sponging miR-217.

This research provides us with some insights in the mechanism between synaptic pathology and circHOMER1. At the same time, such changes are prominent in women, suggesting that we can develop gender-specific diagnostic markers and personalized treatment regimens.

#### 2.4.2 CircRIMS2 and Synaptic Plasticity

CircRIMS2 is elevated in the hippocampus of 4-month-old APP/PS1 mice and its overexpression leads to synaptic dysfunctions and memory deficits. In addition, circRIMS2 is co-localized with miR-3968 in the cytoplasm of N2a cells and inhibits miR-3968 expression by acting as a miRNA sponge. METTL3-dependent N6-methyladenosine (m6A) modifications have been implicated in circRNA biological function and might enhance the degradation or stability of certain circRNAs [54]. In APP/PS1 mice, the expression of METTL3-dependent m6A of circRIMS2 is markedly up-regulated, which ultimately mediates the elevation of circRIMS2 [55].

Ubiquitin-conjugating enzyme E2K (UBE2K) is a promising target of miR-3968 and directly interacts with GluN2B to mediate its degradation. Overexpression of miR-3968 or blocking UBE2K significantly rescues synaptic and memory deficits in AD mice. CircRIMS2 can promote UBE2K-mediated GluN2B degradation by sponging miR-3968, causing memory and synaptic damage in mice. Furthermore, silencing of METTL3 decreases circRIMS2 expression and improves synaptic and cognitive deficits in APP/PS1 mice.

#### 2.4.3 Circ-Vps41 and Synaptic Plasticity

In HT 22 cells, overexpression of circ-Vps41 improves synaptic plasticity and reduces oxidative stress.

Nuclear factor erythroid-2-related factor 2 (Nrf2) is a necessary transcription factor in cyto-antioxidant response and directly interacts with the Vps41 promoter to promote circ-Vps41 expression [56].

Moreover, circ-Vps41 and calcium/calmodulin-dependent protein kinase IV (CaMKIV) mRNA 3’UTR have potential binding sites for miR-26a-5p, and activation of circ-Vps41 by Nrf2 suppresses miR-26a-5p’s binding to CaMKIV through sponging miR-26a-5p, thereby promotes CaMKIV expression. CaMKIV is a key catalytic enzyme for the phosphorylation of transcription factor cAMP-response element binding protein (CREB), and CaMKIV/CREB signal can attenuate neuronal damage and cognitive deficits [57].

In addition, circ-Vps41 is markedly reduced in ageing models induced by D-galactose. Circ-Vps41 overexpression ameliorates the expression of synaptophysin (Syp), an abundant synaptic vesicle membrane protein, increases the density of dendritic spines and alleviates learning and memory impairment related to aging. Circ-Vps41 has a target site of miR-24-3p and negatively regulates miR-24-3p [58], which can target Syp 3'UTR.

In conclusion, circ-Vps41 increases CaMKIV expression by sponging to miR-26a-5p and upregulates Syp by sponging to miR-24-3p, finally improving the learning and memory capacity in aging models.

#### 2.4.4 CircGRIA1 and Synaptic Plasticity

CircGRIA1, a conservative circRNA isoform derived from the genomic loci of AMPA receptor subunit Gria1, is overexpressed in the hippocampus and prefrontal cortex of rhesus macaque, which is related to age and specific in males. In addition, circGRIA1 can negatively regulate the density of synapsin-I components, and circGRIA1 knockdown significantly increases synapsin-I levels.

CircGRIA1 is mainly located in the nucleus and can negatively regulate the transcriptional activity of Gria1 through competitive association with the Gria1 promoter region on 5’-UTR [59]. Gria1 encodes the glutamate receptor α-mino-3-hydroxy-5-methyl-4-isoxazole propionic acid (AMPA) subunit GluR1, and AMPA and N-methyl-D-aspartate (NMDA) receptor-mediated neuronal activity is essential for maintaining synaptic plasticity. High expression of circGRIA1 can reduce not only Gria1 mRNA but also GluR1 protein, thereby diminishing synaptic plasticity.

CircGRIA1 also reduces the mini-excitatory postsynaptic current (mEPSC), which is used to mimic AMPA and NMDA receptor-mediated neuronal activity, further demonstrating the reduction in synaptic plasticity. Synaptic plasticity deficits are strongly associated with disturbances in calcium homeostasis, and knockdown of circGRIA1 can reverse the reduction of calcium, suggesting that it may exacerbate calcium metabolism disorders.

#### 2.4.5 HuD-circRNAs and Synaptic Plasticity

Neuronal RNA-binding protein (RBP) HuD (also known as ELAVL4) participates in various phases of differentiation and maturation of neurons [60]. Most circRNAs bound to HuD have been implicated in neurological development and function. It is previously shown that neuronal RBP HuD can bind and regulate the synaptic localization of circHomer1a, which is related to synaptic plasticity [61]. And HuD overexpressed mice have a 4-fold increase in circUpf2 level in synaptosomes, which can encode plasticity-related proteins.

In a recent study, five circRNAs are found to bind HuD in the dataset: circBrwd1, circFoxp1, circMap1a, circMagi1, and circLppr4, which can regulate regeneration and development of neuron and synaptic plasticity [62], The specific functions of these circRNAs have not been investigated but they are likely to be involved in synaptic plasticity and neural function: Brwd1 is associated with neurodevelopmental dysfunction and AD [63]; CircFoxp1 can regulate the expression of miR-125a-5p, which is associated with the delay of ageing [64]; Map1a is highly expressed in the cytosol and dendrites of neurons in the mammalian brain, and its absence leads to the death of neurons [65]. CircMagi1 encodes a member of the membrane-associated guanylate kinase (MAGUK) family, which is relatively enriched in synaptic membranes [66]. CircLppr4 may be related to lipid phosphate phosphatase-related protein (LPPR) to induce membrane protrusion and synapse growth in neurons [67].

### 2.5 circRNA and Apoptosis/Autophagy

In the autopsy of AD patients, large numbers of apoptotic neurons have been found in the hippocampus and cerebral cortex. Aβ, tau, and inflammation can trigger the apoptotic pathway leading to neuronal death. Inhibition of apoptosis has potential efficacy for early prevention of neuronal death [68]. Autophagy is a vital pathway of degradation that removes aberrant protein accumulations and also is related to the generation and metabolism of tau protein and Aβ. Autophagy dysfunction has been found to happen in the early stages of AD and might promote the progression of AD [69].

#### 2.5.1 Circ-0000950 and Apoptosis

Circ-0000950 overexpression has been shown to promote neuronal apoptosis and suppress neurite outgrowth in cellular AD models, whereas circ-0000950 knockdown enhances neurite outgrowth and decreases neuronal apoptosis. In addition, miR-103 is identified as a direct target of circ-0000950 [70].

MiR-103 has been found to enhance neurite growth and inhibit apoptosis in AD cellular models *via* targeting prostaglandin-endoperoxide synthase 2 (PTGS 2), which is involved in inflammation [71]. Therefore, by sponging miR-103, circ-0000950 can increase inflammatory cytokines and promote apoptosis in AD, providing a circ-0000950/miR-103/PTGS 2 pathway for apoptosis regulation.

These data suggests that the pathological mechanisms of AD do not exist independently, but rather reinforce or constrain each other. CircRNAs may play a pivotal role between them.

#### 2.5.2 Circ-0003611 and Apoptosis

Circ-0003611 is overexpressed in the serum of AD individuals compared with the healthy group. With the increase of Aβ concentration, the levels of circ-0003611 are abnormally increased. Furthermore, treatment of Aβ significantly induces apoptosis, whereas circ-0003611 downregulation can reverse it.

MiR-383-5p is one of the targets of circ-0003611 and is reduced in the serum of AD individuals. Circ-0003611 can reduce miR-383-5p expression, and increase apoptosis and neuronal injury triggered by Aβ [72]. Furthermore, miR-383-5p directly targets and inhibits the expression of kinesin family member 1B(KIF1B). KIF1B is closely associated with axonal transport of mitochondria and vesicles in synapses and is significantly elevated in AD.

Thus, downregulation of circ-0003611 can lower the level of apoptosis and ameliorate AD neuronal damage by regulating the miR-383-5p/KIF1B axis, providing novel therapies against AD.

#### 2.5.3 Circ-0002945 and Apoptosis

Endoplasmic reticulum (ER) is required for protein folding and trafficking because of its dynamic structure [73], and overload of Aβ will cause ER stress (ERS), leading to apoptosis of neurons [74, 75].

The expression level of circ-0002945 is significantly higher in AD serum samples and Aβ-stimulated cells. Inhibition of circ-0002945 attenuates the ERS and apoptosis induced by Aβ. Interestingly, miR-431-5p is decreased in AD serum samples and has a binding site with circ-0002945. MiR-431-5p expression is significantly increased in circ-0002945-silenced cells [76].

In APP/PS1 mice brains, miR-431-5p can target TNF-alpha-induced protein 1 (TNFAIP1), inhibit ROS production and Aβ-induced apoptosis *via* RhoB, which can predict neuronal death severity at an early stage and directly modulate apoptotic reactions in neurons [77].

In summary, circ-0002945 is vital in the regulation of neuronal apoptosis and ERS induced by Aβ *via* interaction with miR-431-5p to regulate TNFAIP1.

#### 2.5.4 CircLPAR1 and Apoptosis

CircRNA lysophosphatidic acid receptor 1 (circLPAR1) is elevated in the serum of AD individuals, and circLPAR1 downregulation suppresses Aβ-induced neuronal apoptosis, oxidative stress and inflammation [78].

Bioinformatics analysis has indicated that circLPAR1 can sponge to miR-212-3p, which is decreased in temporal cortex samples of AD [79] and suppresses neuronal apoptosis by promoting the signaling pathway of PI3K/AKT [80].

MiR-212-3p is identified to target zinc finger protein 217 (ZNF217) and inhibits its expression, which is one of the Krüppel-like family and contributes to Aβ-induced neurotoxicity [81]. Overexpression of ZNF217 reverses the reduction in apoptosis caused by miR-212-3p. Thus, circLPAR1 increases the apoptosis, oxidative stress and inflammation in Aβ-treatment cells through the miR-212-3p / ZNF217 axis.

#### 2.5.5 CircNF1-419 and Autophagy

CircNF1-419 overexpression significantly upregulates the levels of LC3A I, LC3A II, LC3B I, and LC3B II in rat astrocytes, which are autophagy biomarkers. In addition, the results of transmission electron microscopy show the formation of phagosomes, endosomes, autophagosomes, autolysosomes and lysosomes in astrocytes transfected with circNF1-419, suggesting that circNF1-419 promotes autophagy. Moreover, circNF1-419 modulates autophagy *via* the PI3K-I/Akt-AMPK-mTOR signaling pathway [82].

On the other hand, circNF1-419 directly binds to Dynamin-1 and Adaptor protein 2 B1(AP2B1) to induce autophagy and attenuate the pathogenesis of AD, including Aβ1-42, p-Tau/Tau and APOE. Dynamin-1 is indispensable for vesicle formation, especially in receptor-mediated endocytic effects, synaptic vesicle cycling and so on [83]. AP2B1 is an essential participant in clathrin-relative endocytosis and degrades Aβ through autophagy [84].

## 3 CircRNAs AS BIOMARKERS OF AD

Emerging studies have demonstrated that blood biomarkers including circRNAs become attractive tools for AD diagnosis and treatment. CircRNAs have a stable circular structure and can be easily detected in plasma. In addition, the high conservation makes the relative comparison of circRNAs in animal and human models more convenient [85]. Moreover, circRNAs are abundant in the central nervous system and gradually increase as the brain ages, showing sensitivity to neurodegenerative illnesses. Most plasma circRNAs originate from the CNS, thus, theoretically, existing techniques can even identify specific brain regions from which they originate [86]. It has been found that circRNA expression is altered before the onset of substantial symptoms, which means that circRNAs can serve as potential biomarkers for pre-symptomatic and symptomatic AD [87].

### 3.1 circ-0003391

The level of circ-0003391 is lowered in AD individuals' blood samples. And the level of circ-0003391 is positively associated with MoCA, MMSE, RAVLT-I and RAVLT-D scores, and negatively with CDR scores. The results of MRI show that patients with AD exhibit significant atrophy of the hippocampus compared to age-matched healthy people, and circ-0003391 expression is positively associated with volumes of the hippocampus, indicating that some clinical manifestations of AD is notably associated with downregulation of circ-0003391.

In addition, circ-0003391 is specifically decreased in AD individuals in comparison to that in Lewy body dementia (DLB) and vascular dementia (VD) groups, suggesting that circ-0003391 might be used as a potential biomarker to distinguish different types of dementia [88].

As for the mechanism, miR-574-5p may be a possible target of circ-0003391 in AD patients. MiR-574-5p is an important downstream effector of APP-mediated cell cycle progression and neuronal proliferation. APP inhibits neural differentiation by antagonizing the miR-574-5p function [89]. However, the mechanism of circ-0003391/ miR-574-5p requires further research in the future.

### 3.2 CircRNA-050263, circRNA-403959, circRNA-003022, circRNA-10083749, circRNA-102049 and circRNA-102619

The levels of circRNA-050263, circRNA-403959, circRNA-003022 and circRNA-10083749 are upregulated in AD and MCI participants’ plasma samples, while circRNA-102049 and circRNA-102619 expression are downregulated in AD individuals. It is interesting to find that the upregulation of circRNA-403959 is significant in MCI patients compared to AD and controls [90]. This suggests that circRNAs might differentiate the MCI and AD patients.

To further define the diagnostic precision of these six circRNAs as potential biomarkers, ROC curve analyses are performed. The ROC curve analysis shows that circRNA-050263 can accurately distinguish healthy controls from AD patients. CircRNA-102619, circRNA-102049 and circRNA-10083749 have good accuracy, while circRNA-003022 and circRNA-403959 have comparable accuracy.

### 3.3 6-circRNA Panel

CircRNAs have been shown to differentiate AD from other types of dementia and individuals with normal cognitive levels.

For example, circRNA-0077001, circRNA-0022417, circRNA-0014356, circRNA-0014353, and circRNA-0074533 levels are elevated, while circRNA-0089894 is decreased in the blood samples of AD patients. Moreover, these circRNAs are not altered in patients with vascular dementia (VaD), behavioural variability frontotemporal dementia (bvFTD), Parkinson's disease dementia (PDD) and Lewy body dementia (DLB), suggesting that they are probably AD-specific circRNAs [91]. Subsequent ROC curve analysis shows that the combination of these six circRNAs has a higher diagnostic ability than that of a single circRNA. The results of Gene ontology (GO) show that these 6 circRNAs are involved in metabolic processes, immune system processes and synaptic activity associated with AD [92, 93].

### 3.4 CircRNA-001481 and circRNA-000479

Subjective cognitive decline (SCD) is a cognitive disorder that occurs in the early stage of AD [94]. Some circRNAs, including circRNA-001481 and circRNA-000479, are differentially expressed in SCD, aMCI, and nomal controls (NC) samples. CircRNA-001481 is elevated in both SCD and aMCI groups, and it is 1.51-fold higher in the SCD group compared to the aMCI group. In addition, the level of circRNA-000479 in the SCD group has a 1.9-fold increase compared to that in the control group and a 2.2-fold increase compared to that in the aMCI group. These results indicate their promising role in biomarkers for differentiating the SCD group from other groups.

The 3’UTR region of circRNA-001481 is found to have binding sites for miR-1252-5p, miR-4644 and miR-548, and can significantly reduce their expressions, ultimately regulating the embigin expression, which is important for long-term memory [95]. CircRNA-000479 may bind and act as a sponge for miR-942-5p, miR-4753-3p and miR-6739-3p to regulate EPSTI1 expression, which is a potential biomarker for early detection of VaD and AD [96].

CircRNA-001481 and circRNA-000479 may serve as a non-invasive way of screening tests in early AD patients [97]. However, the specific mechanisms need further research.

### 3.5 Circ-AXL, circ-PCCA and circ-GPHN

Ten dysregulated circRNAs are identified in 80 AD individuals, and these circRNAs are abundant in neuronal cell death, inflammation and neurodegenerative pathways. Circ-AXL and circ-GPHN are overexpressed in CSF of AD individuals compared to that in control groups, while the level of circ-PCCA is significantly declined. Further analyses show that the levels of circ-AXL and circ-GPHN forecast a high risk of AD, while circ-PCCA forecast a low risk of AD, and they are independent predictors of the disease risk. Moreover, circ-AXL and circ-GPHN are negatively correlated with MMSE scores, while circ-PCCA is positively correlated with MMSE scores. As for the pathological mechanisms, circ-AXL is positively correlated with both p-tau and t-tau, and circ-GPHN is positively correlated only with t-tau. Circ-AXL is negatively correlated with Aβ42, while circ-PCCA is positively correlated with Aβ42. For the study of the mechanisms, circ-AXL reduces its parental gene AXL transcription, which suppresses inflammation and reduces the apoptotic neuronal cells and debris clearance in the CNS [98]. As for circ-GPHN, it represses gene gephyrin (GPHN) transcription, leading to functional synapse lost, toxic metabolites accumulation and neuroinflammation [99]. Thus, their high expressions correlate with elevated disease severity in AD patients. Circ-PCCA may sponge miR-138-5 to inhibit the activation of glycogen synthase kinase-3β and phosphorylation of tau, therefore, its high expression is associated with remission of disease severity in AD patients [37].

### 3.6 CircRNA KIAA1586

AD-associated circRNA-miRNA-mRNA competition network (ADcirCeNET) show that circRNA KIAA1586 is most frequently found in AD-risk circRNA-associated ceRNAs and has the strongest correlation with AD risk.

CircRNA KIAA1586 can competitively bind to hsa-miR-29b, hsa-miR-101 and hsa-miR-15a, all of which are related to AD. Has-miR-29b is reduced in AD patients [100], suppression of has-miR-101 augments APP levels and influences Aβ accumulation [101], and has-miR-15a is significantly altered in AD brain and predicted to modulate APP [102]. Further, GO analysis shows that circRNA KIAA1586-associated mRNAs are notably enriched in biological processes known to be involved in AD, such as the Wnt signaling pathway, stress-activated MAPK cascade, ensheathment of neurons, and autophagy.

## 4 PERSPECTIVES OF CircRNA IN AD

This review summarizes the biological mechanisms of circRNAs in the development of AD, which will greatly strengthen the comprehension of AD and offer alternative targets for its diagnosis. The current profile of differentially expressed circRNAs in AD is gradually improving, but the specific functions and mechanisms of key circRNAs directly related to AD are still very poorly understood. As far, the understanding of circRNAs is only the tip of the iceberg, and there are majority of annotated circRNAs awaiting functional characterization.

There are many challenges in this field. Firstly, concentrations of most circRNAs are relatively low because reverse clipping is less efficient than typical clipping [103]. Several regulatory factors have been uncovered to ameliorate circRNAs biogenesis, which include intron complementary sequences (ICSs) in flanking introns of circle-forming exons, RBPs and Alu elements [104], thereby promoting reverse splicing, implying that circRNA expression can be regulated by mechanisms of these molecules. Technically, more efforts are needed to improve the detection methods of circRNA. Secondly, the sequence of circRNA completely overlaps with its parental linear RNA transcript [105], and dissecting the biological roles of circRNA is a challenge that requires specific molecular tools to regulate endogenous circRNA activity. Thirdly, emerging studies have only been conducted in a single centre, thus, the sensibility and particularity of chosen circRNAs are inadequate, requiring screening and confirmation in multicenter and large-scale trials [106].

The ultimate goal of investigating the molecular mechanisms of circRNA is to develop circRNA-based diagnosis tools and therapeutic strategies. However, there have been no circRNA-associated treatments for AD in clinical trials yet, which suggests that the efficacy and safety of circRNA-based therapy require further studies. Furthermore, circRNA Drug delivery is indispensable for its therapeutic role in the nervous system. Currently, the most studied circRNA delivery systems are exosomes, viral vectors and nanoparticles, while their targeting accuracy, dosage control, production standardization and immune response still need to be further explored [107].

## CONCLUSION

In our review, multiple circRNAs are correlated with AD and are crucial in pathological mechanisms of AD, such as amyloid production, Tau hyperphosphorylation, neuroinflammation, synapse plasticity, autophagy and apoptosis. Moreover, circRNAs may be used as biomarkers for presymptomatic and symptomatic AD. However, the biological properties and potential mechanisms of circRNAs during AD progress require deeper exploration. There is no doubt that further research on circRNAs will make a great contribution to the clinical diagnosis and therapy for AD.

## LIST OF ABBREVIATIONS

AD: Alzheimer's Disease
APP: Amyloid Precursor Protein
B1N1: Bridging Integrator 1
CDR: Clinical Dementia Score
circRNAs: Circular RNAs
CSF: Cerebrospinal Fluid
FGF7: Fibroblast Growth Factor 7
GO: Gene Ontology
GSK3-β: Glycogen Synthase Kinase-3β
MCI: Mild Cognitive Impairment
MS: Mass Spectroscopy
NFTs: Neurofibrillary Tangles
PHF: Paired Helical Filaments
SCD: Subjective Cognitive Decline
VaD: Vascular Dementia

## References

[1] (2023). 2023 Alzheimer’s disease facts and figures.. Alzheimers Dement..

[2] Andrade-Guerrero J., Santiago-Balmaseda A., Jeronimo-Aguilar P., Vargas-Rodríguez I., Cadena-Suárez A.R., Sánchez-Garibay C., Pozo-Molina G., Méndez-Catalá C.F., Cardenas-Aguayo M.C., Diaz-Cintra S., Pacheco-Herrero M., Luna-Muñoz J., Soto-Rojas L.O. (2023). Alzheimer’s disease: An updated overview of its genetics.. Int. J. Mol. Sci..

[3] Karch C.M., Goate A.M. (2015). Alzheimer’s disease risk genes and mechanisms of disease pathogenesis.. Biol. Psychiatry.

[4] Van Cauwenberghe C., Van Broeckhoven C., Sleegers K. (2016). The genetic landscape of Alzheimer disease: Clinical implications and perspectives.. Genet. Med..

[5] Farrer L.A., Cupples L.A., Haines J.L., Hyman B., Kukull W.A., Mayeux R., Myers R.H., Pericak-Vance M.A., Risch N., van Duijn C.M. (1997). Effects of age, sex, and ethnicity on the association between apolipoprotein E genotype and Alzheimer disease. A meta-analysis.. JAMA.

[6] Gambhir I.S., Misra A., Chakrabarti S.S. (2018). New genetic players in late-onset Alzheimer’s disease: Findings of genome-wide association studies.. Indian J. Med. Res..

[7] Hardy J.A., Higgins G.A. (1992). Alzheimer’s disease: The amyloid cascade hypothesis.. Science.

[8] McLean C.A., Cherny R.A., Fraser F.W., Fuller S.J., Smith M.J., Konrad V., Bush A.I., Masters C.L. (1999). Soluble pool of Aβ amyloid as a determinant of severity of neurodegeneration in Alzheimer’s disease.. Ann. Neurol..

[9] Hyman B.T. (2011). Amyloid-dependent and amyloid-independent stages of Alzheimer disease.. Arch. Neurol..

[10] Querfurth H.W., LaFerla F.M. (2010). Alzheimer’s disease.. N. Engl. J. Med..

[11] You X., Vlatkovic I., Babic A., Will T., Epstein I., Tushev G., Akbalik G., Wang M., Glock C., Quedenau C., Wang X., Hou J., Liu H., Sun W., Sambandan S., Chen T., Schuman E.M., Chen W. (2015). Neural circular RNAs are derived from synaptic genes and regulated by development and plasticity.. Nat. Neurosci..

[12] Chen L.L. (2020). The expanding regulatory mechanisms and cellular functions of circular RNAs.. Nat. Rev. Mol. Cell Biol..

[13] D’Ambra E., Capauto D., Morlando M. (2019). Exploring the regulatory role of circular RNAs in neurodegenerative disorders.. Int. J. Mol. Sci..

[14] Liu K.S., Pan F., Mao X.D., Liu C., Chen Y.J. (2019). Biological functions of circular RNAs and their roles in occurrence of reproduction and gynecological diseases.. Am. J. Transl. Res..

[15] Bach D.H., Lee S.K., Sood A.K. (2019). Circular RNAs in cancer.. Mol. Ther. Nucleic Acids.

[16] Schneider T., Hung L.H., Schreiner S., Starke S., Eckhof H., Rossbach O., Reich S., Medenbach J., Bindereif A. CircRNAprotein
complexes: IMP3 protein component defines subfamily of
circRNPs. 2016, Scientific reports.

[17] Li Z., Huang C., Bao C., Chen L., Lin M., Wang X., Zhong G., Yu B., Hu W., Dai L., Zhu P., Chang Z., Wu Q., Zhao Y., Jia Y., Xu P., Liu H., Shan G. (2015). Exon-intron circular RNAs regulate transcription in the nucleus.. Nat. Struct. Mol. Biol..

[18] Du W.W., Fang L., Yang W., Wu N., Awan F.M., Yang Z., Yang B.B. (2017). Induction of tumor apoptosis through a circular RNA enhancing Foxo3 activity.. Cell Death Differ..

[19] Chen C.K., Cheng R., Demeter J., Chen J., Weingarten-Gabbay S., Jiang L., Snyder M.P., Weissman J.S., Segal E., Jackson P.K., Chang H.Y. (2021). Structured elements drive extensive circular RNA translation.. Mol. Cell.

[20] Legnini I., Di Timoteo G., Rossi F., Morlando M., Briganti F., Sthandier O., Fatica A., Santini T., Andronache A., Wade M., Laneve P., Rajewsky N., Bozzoni I. (2017). Circ-ZNF609 is a circular RNA that can be translated and functions in myogenesis.. Mol. Cell.

[21] Gruner H., Cortés-López M., Cooper D.A., Bauer M., Miura P. (2016). CircRNA accumulation in the aging mouse brain.. Sci. Rep..

[22] Cogswell J.P., Ward J., Taylor I.A., Waters M., Shi Y., Cannon B., Kelnar K., Kemppainen J., Brown D., Chen C., Prinjha R.K., Richardson J.C., Saunders A.M., Roses A.D., Richards C.A. (2008). Identification of miRNA changes in Alzheimer’s disease brain and CSF yields putative biomarkers and insights into disease pathways.. J. Alzheimers Dis..

[23] Lu Y., Tan L., Wang X. (2019). Circular HDAC9/microRNA-138/sirtuin-1 pathway mediates synaptic and amyloid precursor protein processing deficits in Alzheimer’s disease.. Neurosci. Bull..

[24] Mo D., Li X., Raabe C.A., Rozhdestvensky T.S., Skryabin B.V., Brosius J. (2020). Circular RNA encoded amyloid beta peptides-A novel putative player in Alzheimer’s disease.. Cells.

[25] Urdánoz-Casado A., Sánchez-Ruiz de Gordoa J., Robles M., Roldan M., Macías Conde M., Acha B., Blanco-Luquin I., Mendioroz M. (2023). circRNA from APP gene changes in Alzheimer’s disease human brain.. Int. J. Mol. Sci..

[26] Riancho J., Vázquez-Higuera J.L., Pozueta A., Lage C., Kazimierczak M., Bravo M., Calero M., Gonalezález A., Rodríguez E., Lleó A., Sánchez-Juan P. (2017). MicroRNA profile in patients with Alzheimer’s disease: Analysis of miR-9-5p and miR-598 in raw and exosome enriched cerebrospinal fluid samples.. J. Alzheimers Dis..

[27] Song C., Zhang Y., Huang W., Shi J., Huang Q., Jiang M., Qiu Y., Wang T., Chen H., Wang H. (2022). Circular RNA Cwc27 contributes to Alzheimer’s disease pathogenesis by repressing Pur-α activity.. Cell Death Differ..

[28] Li N., Zhang D., Guo H., Yang Q., Li P., He Y. (2022). Inhibition of circ_0004381 improves cognitive function via miR-647/PSEN1 axis in an Alzheimer disease mouse model.. J. Neuropathol. Exp. Neurol..

[29] Chen H.H., Eteleeb A., Wang C., Fernandez M.V., Budde J.P., Bergmann K., Norton J., Wang F., Ebl C., Morris J.C., Perrin R.J., Bateman R.J., McDade E., Xiong C., Goate A., Farlow M., Chhatwal J., Schofield P.R., Chui H., Harari O., Cruchaga C., Ibanez L. (2022). Circular RNA detection identifies circPSEN1 alterations in brain specific to autosomal dominant Alzheimer’s disease.. Acta Neuropathol. Commun..

[30] García-Escudero V., Gargini R., Martín-Maestro P., García E., García-Escudero R., Avila J. (2017). Tau mRNA 3′UTR-to-CDS ratio is increased in Alzheimer disease.. Neurosci. Lett..

[31] Braak H., Braak E. (1991). Neuropathological stageing of Alzheimer-related changes.. Acta Neuropathol..

[32] Macedo A.C., Tissot C., Therriault J., Servaes S., Wang Y.T., Fernandez-Arias J., Rahmouni N., Lussier F.Z., Vermeiren M., Bezgin G., Vitali P., Ng K.P., Zimmer E.R., Guiot M.C., Pascoal T.A., Gauthier S., Rosa-Neto P. (2023). The use of tau PET to stage Alzheimer disease according to the braak staging framework.. J. Nucl. Med..

[33] Welden J.R., Margvelani G., Arizaca Maquera K.A., Gudlavalleti B., Miranda Sardón S.C., Campos A.R., Robil N., Lee D.C., Hernandez A.G., Wang W.X., Di J., de la Grange P., Nelson P.T., Stamm S. (2022). RNA editing of microtubule-associated protein tau circular RNAs promotes their translation and tau tangle formation.. Nucleic Acids Res..

[34] Welden J.R., Stamm S. (2019). Pre-mRNA structures forming circular RNAs.. Biochim. Biophys. Acta. Gene Regul. Mech..

[35] George C. X., Gan Z., Liu Y., Samuel C. E. (2011). Adenosine deaminases acting on RNA, RNA editing, and interferon action.. J. Interferon Cytokine Res..

[36] Zhang Q., Chen B., Yang P., Wu J., Pang X., Pang C. (2022). Bioinformatics-based study reveals that AP2M1 is regulated by the circRNA-miRNA-mRNA interaction network and affects Alzheimer’s disease.. Front. Genet..

[37] Wang X., Tan L., Lu Y., Peng J., Zhu Y., Zhang Y., Sun Z. (2015). MicroRNA‐138 promotes tau phosphorylation by targeting retinoic acid receptor alpha.. FEBS Lett..

[38] Li Y., Fan H., Sun J., Ni M., Zhang L., Chen C., Hong X., Fang F., Zhang W., Ma P. (2020). Circular RNA expression profile of Alzheimer’s disease and its clinical significance as biomarkers for the disease risk and progression.. Int. J. Biochem. Cell Biol..

[39] Puri S., Hu J., Sun Z., Lin M., Stein T.D., Farrer L.A., Wolozin B., Zhang X. (2023). Identification of circRNAs linked to Alzheimer’s disease and related dementias.. Alzheimers Dement..

[40] Luo M., Zeng Q., Jiang K., Zhao Y., Long Z., Du Y., Wang K., He G. (2022). Estrogen deficiency exacerbates learning and memory deficits associated with glucose metabolism disorder in APP/PS1 double transgenic female mice.. Genes Dis..

[41] Arizaca Maquera K.A., Welden J.R., Margvelani G., Miranda Sardón S.C., Hart S., Robil N., Hernandez A.G., de la Grange P., Nelson P.T., Stamm S. (2023). Alzheimer’s disease pathogenetic progression is associated with changes in regulated retained introns and editing of circular RNAs.. Front. Mol. Neurosci..

[42] Cai Z., Yan L.J., Ratka A. (2013). Telomere shortening and Alzheimer’s disease.. Neuromol. Med..

[43] Xu X., Gu D., Xu B., Yang C., Wang L. (2022). Circular RNA circ_0005835 promotes promoted neural stem cells proliferation and differentiate to neuron and inhibits inflammatory cytokines levels through miR-576-3p in Alzheimer’s disease.. Environ. Sci. Pollut. Res. Int..

[44] Li Y., Han X., Fan H., Sun J., Ni M., Zhang L., Fang F., Zhang W., Ma P. (2022). Circular RNA AXL increases neuron injury and inflammation through targeting microRNA-328 mediated BACE1 in Alzheimer’s disease.. Neurosci. Lett..

[45] Meng S., Wang B., Li W. (2022). CircAXL knockdown alleviates Aβ1-42-induced neurotoxicity in Alzheimer’s disease via repressing PDE4A by releasing miR-1306-5p.. Neurochem. Res..

[46] Wang R., Zhang J. (2020). Clinical significance of miR-433 in the diagnosis of Alzheimer’s disease and its effect on Aβ-induced neurotoxicity by regulating JAK2.. Exp. Gerontol..

[47] Xu W., Li K., Fan Q., Zong B., Han L. (2020). Knockdown of long non-coding RNA SOX21-AS1 attenuates amyloid-β-induced neuronal damage by sponging miR-107.. Biosci. Rep..

[48] Zeng C., Xing H., Chen M., Chen L., Li P., Wu X., Li L. (2022). Circ_0049472 regulates the damage of Aβ-induced SK-N-SH and CHP-212 cells by mediating the miR-107/KIF1B axis.. Exp. Brain Res..

[49] Melo T.Q., D’unhao A.M., Martins S.A., Farizatto K.L.G., Chaves R.S., Ferrari M.F.R. (2013). Rotenone-dependent changes of anterograde motor protein expression and mitochondrial mobility in brain areas related to neurodegenerative diseases.. Cell. Mol. Neurobiol..

[50] Xiong W., Li D., Feng Y., Jia C., Zhang X., Liu Z. (2023). CircLPAR1 promotes neuroinflammation and oxidative stress in APP/PS1 mice by inhibiting SIRT1/Nrf-2/HO-1 axis through destabilizing GDF-15 mRNA.. Mol. Neurobiol..

[51] Koh M.T., Haberman R.P., Foti S., McCown T.J., Gallagher M. (2010). Treatment strategies targeting excess hippocampal activity benefit aged rats with cognitive impairment.. Neuropsychopharmacology.

[52] Dejanovic B., Sheng M., Hanson J.E. (2024). Targeting synapse function and loss for treatment of neurodegenerative diseases.. Nat. Rev. Drug Discov..

[53] Urdánoz-Casado A., Sánchez-Ruiz de Gordoa J., Robles M., Acha B., Roldan M., Zelaya M.V., Blanco-Luquin I., Mendioroz M. (2021). Gender-dependent deregulation of linear and circular RNA variants of HOMER1 in the entorhinal cortex of Alzheimer’s disease.. Int. J. Mol. Sci..

[54] Zhang L., Hou C., Chen C., Guo Y., Yuan W., Yin D., Liu J., Sun Z. (2020). The role of N6-methyladenosine (m6A) modification in the regulation of circRNAs.. Mol. Cancer.

[55] Wang X., Xie J., Tan L., Lu Y., Shen N., Li J., Hu H., Li H., Li X., Cheng L. (2023). N6-methyladenosine-modified circRIMS2 mediates synaptic and memory impairments by activating GluN2B ubiquitination in Alzheimer’s disease.. Transl. Neurodegener..

[56] Zhang R., Gao Y., Li Y., Geng D., Liang Y., He Q., Wang L., Cui H. (2022). Nrf2 improves hippocampal synaptic plasticity, learning and memory through the circ-Vps41/miR-26a-5p/CaMKIV regulatory network.. Exp. Neurol..

[57] Kotera I., Sekimoto T., Miyamoto Y., Saiwaki T., Nagoshi E., Sakagami H., Kondo H., Yoneda Y. (2005). Importin α transports CaMKIV to the nucleus without utilizing importin β.. EMBO J..

[58] Li Y., Wang H., Gao Y., Zhang R., Liu Q., Xie W., Liu Z., Geng D., Wang L. (2022). Circ-Vps41 positively modulates Syp and its overexpression improves memory ability in aging mice.. Front. Mol. Neurosci..

[59] Xu K., Zhang Y., Xiong W., Zhang Z., Wang Z., Lv L., Liu C., Hu Z., Zheng Y.T., Lu L., Hu X.T., Li J. (2020). CircGRIA1 shows an age-related increase in male macaque brain and regulates synaptic plasticity and synaptogenesis.. Nat. Commun..

[60] Oliver R.J., Brigman J.L., Bolognani F., Allan A.M., Neisewander J.L., Perrone-Bizzozero N.I. (2018). Neuronal RNA‐binding protein HuD regulates addiction‐related gene expression and behavior.. Genes Brain Behav..

[61] Zimmerman A.J., Hafez A.K., Amoah S.K., Rodriguez B.A., Dell’Orco M., Lozano E., Hartley B.J., Alural B., Lalonde J., Chander P., Webster M.J., Perlis R.H., Brennand K.J., Haggarty S.J., Weick J., Perrone-Bizzozero N., Brigman J.L., Mellios N. (2020). A psychiatric disease-related circular RNA controls synaptic gene expression and cognition.. Mol. Psychiatry.

[62] Dell’Orco M., Oliver R.J., Perrone-Bizzozero N. (2020). HuD binds to and regulates circular RNAs derived from neuronal development- and synaptic plasticity-associated genes.. Front. Genet..

[63] Quan X., Liang H., Chen Y., Qin Q., Wei Y., Liang Z. (2020). Related network and differential expression analyses identify nuclear genes and pathways in the hippocampus of Alzheimer disease.. Med. Sci. Monit..

[64] Bacon C., Schneider M., Le Magueresse C., Froehlich H., Sticht C., Gluch C., Monyer H., Rappold G.A. (2015). Brain-specific Foxp1 deletion impairs neuronal development and causes autistic-like behaviour.. Mol. Psychiatry.

[65] Liu Y., Lee J.W., Ackerman S.L. (2015). Mutations in the microtubule-associated protein 1A (Map1a) gene cause Purkinje cell degeneration.. J. Neurosci..

[66] Dell’Orco M., Oliver R.J., Perrone-Bizzozero N. (2020). HuD binds to and regulates circular RNAs derived from neuronal development- and synaptic plasticity-associated genes.. Front Genet.

[67] Yu P., Agbaegbu C., Malide D.A., Wu X., Katagiri Y., Hammer J.A., Geller H.M. (2015). Cooperative interactions of LPPR/PRG family members in membrane localization and alteration of cellular morphology.. J. Cell Sci..

[68] Ankarcrona M., Winblad B. (2005). Biomarkers for apoptosis in Alzheimer’s disease.. Int. J. Geriatr. Psychiatry.

[69] Li Q., Liu Y., Sun M. (2017). Autophagy and Alzheimer’s Disease.. Cell. Mol. Neurobiol..

[70] Yang H., Wang H., Shang H., Chen X., Yang S., Qu Y., Ding J., Li X. (2019). Circular RNA circ_0000950 promotes neuron apoptosis, suppresses neurite outgrowth and elevates inflammatory cytokines levels via directly sponging miR-103 in Alzheimer’s disease.. Cell Cycle.

[71] Yang H., Wang H., Shu Y., Li X. (2018). miR-103 promotes neurite outgrowth and suppresses cells apoptosis by targeting prostaglandin-endoperoxide synthase 2 in cellular models of Alzheimer’s disease.. Front. Cell. Neurosci..

[72] Li Y., Wang H., Chen L., Wei K., Liu Y., Han Y., Xia X. (2022). Circ_0003611 regulates apoptosis and oxidative stress injury of Alzheimer’s disease via miR-383-5p/KIF1B axis.. Metab. Brain Dis..

[73] Schwarz D.S., Blower M.D. (2016). The endoplasmic reticulum: Structure, function and response to cellular signaling.. Cell. Mol. Life Sci..

[74] Goswami P., Afjal M.A., Akhter J., Mangla A., Khan J., Parvez S., Raisuddin S. (2020). Involvement of endoplasmic reticulum stress in amyloid β (1-42)-induced Alzheimer’s like neuropathological process in rat brain.. Brain Res. Bull..

[75] Xu T.T., Zhang Y., He J.Y., Luo D., Luo Y., Wang Y.J., Liu W., Wu J., Zhao W., Fang J., Guan L., Huang S., Wang H., Lin L., Zhang S.J., Wang Q. (2018). Bajijiasu ameliorates β-amyloid-triggered endoplasmic reticulum stress and related pathologies in an Alzheimer’s disease model.. Cell. Physiol. Biochem..

[76] Li G., Liang R., Lian Y., Zhou Y. (2022). Circ_0002945 functions as a competing endogenous RNA to promote Aβ25-35-induced endoplasmic reticulum stress and apoptosis in SK-N-SH cells and human primary neurons.. Brain Res..

[77] Xiao Y., Li Y., Zhang H., Yang L., Jiang Y., Wei C., Feng X., Xun Y., Yuan S., Xiang S., Liu N. (2021). TNFAIP1 is upregulated in APP/PS1 mice and promotes apoptosis in SH-SY5Y cells by binding to RhoB.. J Mol Neurosci.

[78] Wu L., Du Q., Wu C. (2021). CircLPAR1/miR-212-3p/ZNF217 feedback loop promotes amyloid β-induced neuronal injury in Alzheimer’s Disease.. Brain Res..

[79] Pichler S., Gu W., Hartl D., Gasparoni G., Leidinger P., Keller A., Meese E., Mayhaus M., Hampel H., Riemenschneider M. (2017). The miRNome of Alzheimer’s disease: Consistent downregulation of the miR-132/212 cluster.. Neurobiol. Aging.

[80] Wang Y., Chang Q. (2020). MicroRNA miR-212 regulates PDCD4 to attenuate Aβ25–35-induced neurotoxicity via PI3K/AKT signaling pathway in Alzheimer’s disease.. Biotechnol. Lett..

[81] Gao Y., Zhang N., Lv C., Li N., Li X., Li W. (2020). lncRNA SNHG1 Knockdown Alleviates Amyloid-β-Induced Neuronal Injury by Regulating ZNF217 via Sponging miR-361-3p in Alzheimer’s Disease.. J. Alzheimers Dis..

[82] Diling C., Yinrui G., Longkai Q., Xiaocui T., Yadi L., Xin Y., Guoyan H., Ou S., Tianqiao Y., Dongdong W., Yizhen X., Yang B.B., Qingping W. (2019). Circular RNA NF1-419 enhances autophagy to ameliorate senile dementia by binding Dynamin-1 and Adaptor protein 2 B1 in AD-like mice.. Aging (Albany NY).

[83] Antonny B., Burd C., De Camilli P., Chen E., Daumke O., Faelber K., Ford M., Frolov V.A., Frost A., Hinshaw J.E., Kirchhausen T., Kozlov M.M., Lenz M., Low H.H., McMahon H., Merrifield C., Pollard T.D., Robinson P.J., Roux A., Schmid S. (2016). Membrane fission by dynamin: What we know and what we need to know.. EMBO J..

[84] Krance S.H., Wu C.Y., Chan A.C.Y., Kwong S., Song B.X., Xiong L.Y., Ouk M., Chen M.H., Zhang J., Yung A., Stanley M., Herrmann N., Lanctôt K.L., Swardfager W. (2022). Endosomal-Lysosomal and Autophagy Pathway in Alzheimer’s Disease: A Systematic Review and Meta-Analysis.. J. Alzheimers Dis..

[85] Zhang Z., Yang T., Xiao J. (2018). Circular RNAs: Promising biomarkers for human diseases.. EBioMedicine.

[86] Akhter R. (2018). Circular RNA and Alzheimer’s disease.. Adv. Exp. Med. Biol..

[87] Dube U., Del-Aguila J.L., Li Z., Budde J.P., Jiang S., Hsu S., Ibanez L., Fernandez M.V., Farias F., Norton J., Gentsch J., Wang F., Allegri R., Amtashar F., Benzinger T., Berman S., Bodge C., Brandon S., Brooks W., Buck J., Buckles V., Chea S., Chrem P., Chui H., Cinco J., Clifford J., D’Mello M., Donahue T., Douglas J., Edigo N., Erekin-Taner N., Fagan A., Farlow M., Farrar A., Feldman H., Flynn G., Fox N., Franklin E., Fujii H., Gant C., Gardener S., Ghetti B., Goate A., Goldman J., Gordon B., Gray J., Gurney J., Hassenstab J., Hirohara M., Holtzman D., Hornbeck R., DiBari S.H., Ikeuchi T., Ikonomovic S., Jerome G., Jucker M., Kasuga K., Kawarabayashi T., Klunk W., Koeppe R., Kuder-Buletta E., Laske C., Levin J., Marcus D., Martins R., Mason N.S., Maue-Dreyfus D., McDade E., Montoya L., Mori H., Nagamatsu A., Neimeyer K., Noble J., Norton J., Perrin R., Raichle M., Ringman J., Roh J.H., Schofield P., Shimada H., Shiroto T., Shoji M., Sigurdson W., Sohrabi H., Sparks P., Suzuki K., Swisher L., Taddei K., Wang J., Wang P., Weiner M., Wolfsberger M., Xiong C., Xu X., Salloway S., Masters C.L., Lee J-H., Graff-Radford N.R., Chhatwal J.P., Bateman R.J., Morris J.C., Karch C.M., Harari O., Cruchaga C. (2019). An atlas of cortical circular RNA expression in Alzheimer disease brains demonstrates clinical and pathological associations.. Nat. Neurosci..

[88] Liu L., Chen X., Chen Y.H., Zhang K. (2020). Identification of circular RNA HSA_Circ_0003391 in peripheral blood is potentially associated with Alzheimer’s disease.. Front. Aging Neurosci..

[89] Zhang W., Thevapriya S., Kim P.J., Yu W.P., Shawn Je H., King Tan E., Zeng L. (2014). Amyloid precursor protein regulates neurogenesis by antagonizing miR-574-5p in the developing cerebral cortex.. Nat. Commun..

[90] Piscopo P., Manzini V., Rivabene R., Crestini A., Le Pera L., Pizzi E., Veroni C., Talarico G., Peconi M., Castellano A.E., D’Alessio C., Bruno G., Corbo M., Vanacore N., Lacorte E. (2022). A plasma circular RNA profile differentiates subjects with Alzheimer’s disease and mild cognitive impairment from healthy controls.. Int. J. Mol. Sci..

[91] Ren Z., Chu C., Pang Y., Cai H., Jia L. (2022). A circular RNA blood panel that differentiates Alzheimer’s disease from other dementia types.. Biomark. Res..

[92] De Strooper B., Karran E. (2016). The Cellular Phase of Alzheimer’s disease.. Cell.

[93] Long J.M., Holtzman D.M. (2019). Alzheimer disease: An update on pathobiology and treatment strategies.. Cell.

[94] Jiang L., Sui D., Qiao K., Dong H.M., Chen L., Han Y. (2018). Impaired functional criticality of human brain during Alzheimer’s disease progression.. Sci. Rep..

[95] Wilson M.C., Meredith D., Fox J.E.M., Manoharan C., Davies A.J., Halestrap A.P. (2005). Basigin (CD147) is the target for organomercurial inhibition of monocarboxylate transporter isoforms 1 and 4: The ancillary protein for the insensitive MCT2 is EMBIGIN (gp70).. J. Biol. Chem..

[96] Luo H., Han G., Wang J., Zeng F., Li Y., Shao S., Song F., Bai Z., Peng X., Wang Y.J., Shi X., Lei H. (2016). Common aging signature in the peripheral blood of vascular dementia and Alzheimer’s disease.. Mol. Neurobiol..

[97] Zheng D., Tahir R.A., Yan Y., Zhao J., Quan Z., Kang G., Han Y., Qing H. (2022). Screening of human circular RNAs as biomarkers for early onset detection of Alzheimer’s disease.. Front. Neurosci..

[98] Weinger J.G., Brosnan C.F., Loudig O., Goldberg M.F., Macian F., Arnett H.A., Prieto A.L., Tsiperson V., Shafit-Zagardo B. (2011). Loss of the receptor tyrosine kinase Axl leads to enhanced inflammation in the CNS and delayed removal of myelin debris during experimental autoimmune encephalomyelitis.. J. Neuroinflammation.

[99] Hales C.M., Rees H., Seyfried N.T., Dammer E.B., Duong D.M., Gearing M., Montine T.J., Troncoso J.C., Thambisetty M., Levey A.I., Lah J.J., Wingo T.S. (2013). Abnormal gephyrin immunoreactivity associated with Alzheimer disease pathologic changes.. J. Neuropathol. Exp. Neurol..

[100] Pereira P.A., Tomás J.F., Queiroz J.A., Figueiras A.R., Sousa F. (2016). Recombinant pre-miR-29b for Alzheimer’s disease therapeutics.. Sci. Rep..

[101] Vilardo E., Barbato C., Ciotti M., Cogoni C., Ruberti F. (2010). MicroRNA-101 regulates amyloid precursor protein expression in hippocampal neurons.. J. Biol. Chem..

[102] Hébert S.S., Horré K., Nicolaï L., Papadopoulou A.S., Mandemakers W., Silahtaroglu A.N., Kauppinen S., Delacourte A., De Strooper B. (2008). Loss of microRNA cluster miR-29a/b-1 in sporadic Alzheimer’s disease correlates with increased BACE1/β-secretase expression.. Proc. Natl. Acad. Sci. USA.

[103] Liu Y., Cheng X., Li H., Hui S., Zhang Z., Xiao Y., Peng W. (2022). Non-coding RNAs as novel regulators of neuroinflammation in Alzheimer’s disease.. Front. Immunol..

[104] Feng X.Y., Zhu S.X., Pu K.J., Huang H.J., Chen Y.Q., Wang W.T. (2023). New insight into circRNAs: Characterization, strategies, and biomedical applications.. Exp. Hematol. Oncol..

[105] Kranick J.C., Chadalavada D.M., Sahu D., Showalter S.A. (2017). Engineering double-stranded RNA binding activity into the Drosha double-stranded RNA binding domain results in a loss of microRNA processing function.. PLoS One.

[106] Yu X., Liu H., Chang N., Fu W., Guo Z., Wang Y. (2023). Circular RNAs: New players involved in the regulation of cognition and cognitive diseases.. Front. Neurosci..

[107] Dong J., Zeng Z., Huang Y., Chen C., Cheng Z., Zhu Q. (2023). Challenges and opportunities for circRNA identification and delivery.. Crit. Rev. Biochem. Mol. Biol..

